# Thin-cap fibroatheroma association with local inflammatory activity in coronary disease: an optical-coherence tomography study

**DOI:** 10.31744/einstein_journal/2025AO1592

**Published:** 2025-10-20

**Authors:** Stefano Garzon, Luiz Fernando Muniz Pinheiro, Felipe Mateus Bezerra, Guy Fernando de Almeida Prado, José Mariani, Willterson Carlos Bandeira, Breno Oliveira Almeida, Pedro Alves Lemos

**Affiliations:** 1 Hospital Israelita Albert Einstein Interventional Cardiology São Paulo SP Brazil Interventional Cardiology, Hospital Israelita Albert Einstein, São Paulo, SP, Brazil.; 2 Sapienza University Department of Clinical and Molecular Medicine Rome Italy Department of Clinical and Molecular Medicine, Sapienza University, Rome, Italy.

**Keywords:** Tomography, optical coherence, Coronary artery disease, Plaque, atherosclerotic, Microphages, Inflammation, Atherosclerosis

## Abstract

**Objective:**

The aim of the present study is to assess whether the intensity of local inflammation relates to the presence of thin-cap fibroatheromas.

**Methods:**

Retrospective, single-center study of patients that underwent optical coherence tomography imaging and had either *de novo* or in-stent neoatherosclerosis. Intensity of macrophage accumulation and volume of neovascularization were measured for all lesions. Logistic binary regressions were used for uni- and multivariate analysis.

**Results:**

A total of the 92 lesions in 84 patients were selected. The degree of macrophage accumulation was higher in thin-cap fibroatheromas than non- thin-cap fibroatheromas lesions (5.0 *versus* 2.17; p<0.01). Neovascularization was more frequent in thin-cap fibroatheromas than non-thin-cap fibroatheromas lesions (87.5% *versus* 65.7%, p=0.04), and thin-cap fibroatheromas had a larger volume of neovascularization than non- thin-cap fibroatheromas plaques (92.2 *versus* 23.0 x 1000*μ*m^3^/mm, p<0.01). At multivariate logistic analysis, neovascularization volume and degree of macrophage accumulation remained independently associated with thin-cap fibroatheromas. The dataset was divided according to the highest tercile of neovascularization volume (≥87.2 x 1000*μ*m^3^/mm) and macrophage accumulation score (≥4.6). Plaques with low levels of neovascularization and macrophages were classified as thin-cap fibroatheromas in 14% of cases. Thin-cap fibroatheromas was present in 61.5% of plaques with high macrophagic and neovascularization content.

**Conclusion:**

Lesions with more macrophage accumulation and higher volumes of neovascularization are more likely to be thin-cap fibroatheromas.

## Highlights

■ Optical coherence tomography enables in vivo characterization of inflammatory activity within coronary plaques.
■ Thin-cap fibroatheromas demonstrate greater macrophage accumulation than non-thin-cap fibroatheromas.
■ Neovascularization is more frequent and quantitatively greater in thin-cap fibroatheromas.
■ The combined burden of macrophages and neovessels provides predictive value for plaque vulnerability.

## In Brief

In Brief Garzon et al. used optical coherence tomography to investigate the relationship between plaque inflammation and thin-cap fibroatheromas. Increased macrophage accumulation and greater neovascularization volume were independently associated with thin-cap fibroatheromas, reflecting an *in vivo* phenotype consistent with highly inflamed and vulnerable plaque.

## Graphical abstract

**Figure:**
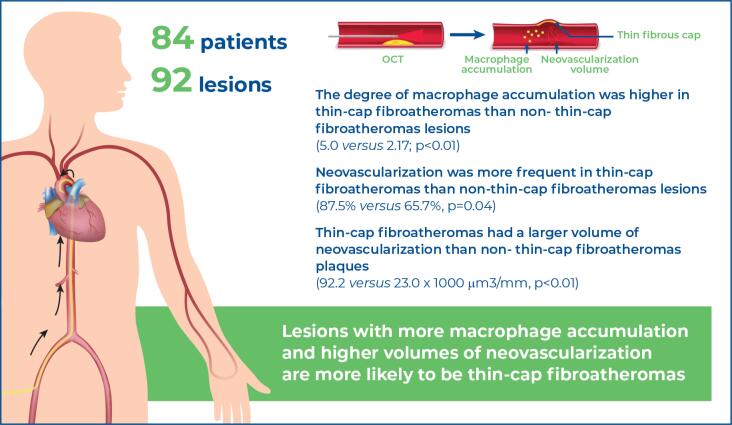

## INTRODUCTION

Inflammation is paramount in the development of atherosclerosis in coronary artery disease (CAD).^(1,2)^ Inflammatory cells modulate local modifications within the arterial wall that ultimately lead to the formation of the so-called atherosclerotic plaque.^(3–5)^ Intra-plaque angiogenesis seems to be a major component of the intricate pathophysiology of inflammation-related plaque development.^(6)^

Intravascular optical coherence tomography (OCT) is an invasive imaging method that has been widely adopted for investigating CAD.^(7,8)^ Due to its near-histology imaging definition, OCT can evaluate plaque components that are practically out of reach for any other intravascular imaging method, such as lipid and calcific tissue component, fibrous cap thickness, intra-plaque neovascularization, and macrophage infiltration.^(9–11)^

Thin-cap fibroatheromas (TCFA) are recognized as markers of plaque vulnerability and their presence increases the risk of plaque rupture and of acute coronary events.^(12,13)^ Previous studies have demonstrated the association between TCFA and inflammation.^(14,15)^ However, there is a paucity of data exploring whether the intensity of inflammation is proportional to the risk of TCFA development.

## OBJECTIVE

To assess whether thin-cap fibroatheromas is associated with the level of local inflammation and its consequences, assessed *in vivo* by the degree of cell infiltration and intra-plaque neovascularization respectively.

## METHODS

### Patient selection

We conducted a search for all patients who underwent coronary OCT in our institution between January of 2012 and December of 2019. Every OCT run performed in that period was reviewed and selected for final analysis if presenting: i) one or more *de novo* atherosclerotic lesions (defined as a plaque arc ≥180°), or ii) one or more neoatherosclerotic lesions (defined as in-stent tissue with calcific or lipidic deposits measuring at least 300*μ*m in thickness). Lesions in the same vessel were considered discrete, and counted as such, if separated by a normal segment longer than 10mm. We only included lesions in which OCT was performed prior to any intervention. Restenotic lesions (*i.e*. in-stent lesions not classified as neo-atherosclerosis) as well as lesions at stent edges (5-mm proximal or distal) were not included for analysis. This study was approved by the local ethics committee of the *Hospital Israelita Albert Eisntein* (CAAE: 18369019500000071; # 3.722.061) and is in accordance with the Declaration of Helsinki.

### Image acquisition and analysis

Image acquisition was performed during injection of contrast media as described elsewhere,^(16,17)^ using a frequency-domain OCT system (C7 or Ilumien OPTIS system, C7 DragonFly or DragonFly II imaging catheters, St. Jude Medical, St. Paul, MN, USA). Off-line quantitative OCT analyses were performed using QIvus 3.0 (Medis Medical, The Netherlands).

Lesions were analyzed according to standard definitions, as suggested elsewhere.^(17)^ Two independent reviewers blinded to any clinical information performed the evaluations of all OCT images. Any disagreement between the reviewers was resolved by consensus. Thin-cap fibroatheromas were defined as regions with a lipid arc >90° and cap thickness <65*μ*m.

The presence of macrophages was defined by signal-rich, distinct, or confluent punctate images exceeding the intensity of background speckle noise. For each frame, the level of macrophage accumulation was semi-quantitatively graded using a score from 0 to 4. The degree of macrophage accumulation for each plaque was calculated as the sum of cross-section grades normalized for the lesion length.^(18)^

Neovascularization was defined as no-signal, intra-plaque structures without connection to the vessel lumen measuring between 50-300*μ*m and recognized in ≥3 consecutive frames.^(19)^ The volume of neovascularization was calculated by summing the area of neovascularization in each frame according to Simpson's rule; finally, the volume of neovascularization was indexed by plaque length.

Quantitative parameters included plaque length, minimum lumen area (MLA), mean lumen cross-sectional area (CSA) reference $\left(\frac{\text{Proximal CSA-Distal CSA}}{2}\right)$ and area stenosis $\left(1-\frac{\text{MLA}}{\text{Mean lumen CSA reference}}x100\right)$.

### Statistical analysis

Statistical analyses were performed using SPSS 26.0 (IBM Corp. Armonk, NY, USA). Categorical variables are presented as counts (percentage) and were analyzed using Chi-square. Continuous variables are presented as median and interquartile range and were analyzed using Mann-Whitney U test. Logistic binary regressions were used for uni- and multivariate analysis. P values <0.05 were defined as statistically significant.

## RESULTS

From a total of 121 patients, 92 lesions from 84 patients were selected for analysis. The median age was 60 years, most patients were male, with hypertension. Almost one-third of our population had diabetes and more than 60% presented with an acute coronary syndrome (Table 1).

**Table 1:** Baseline characteristics

Patients	
Sex (male)	65 (77.4)
Age (years)	60 (55-70)
Hypertension	52 (61.9)
*Diabetes mellitus*	24 (28.6)
Hyperlipidemia	66 (78.6)
Smoker (present or past)	44 (52.4)
Acute coronary syndrome	51 (60.7)

Values are median (interquartile range) or number (%).

Macrophages were ubiquitous and detected in almost all lesions. However, the degree of macrophage accumulation was significantly more pronounced in TCFA than non-TCFA lesions (5.0 [3.7-6.9] *versus* 2.17 [0.8-4.6] respectively; p<0.01), and (Table 2).

**Table 2:** Lesion characteristics on optical coherence tomography

		No TCFA (n=68)	TCFA (n=24)	p value
Plaque type				0.23
	*De novo*	57 (83.8)	17 (70.8)	
	Neoatherosclerosis	11 (16.2)	7 (29.2)	
Plaque length (mm)		22.7 (16.9-31.9)	30.2 (17.9-40.5)	0.13
Area stenosis (%)		65.2 (53.8-73.2)	70.0 (57.2-75.9)	0.25
MLA (mm2)		2.5 (1.5-3.3)	2.0 (1.5-3.6)	0.63
Neovascularization		44 (65.7)	21 (87.5)	0.04
Neovascularization volume		23.0 (0.0-99.0)	92.2 (31.8-239.7)	<0.01
Macrophage		65 (95.6)	24 (100.0)	0.56
Macrophage accumulation		2.17 (0.8-4.6)	5.0 (3.7-6.9)	<0.01
Plaque rupture		5 (7.6)	12 (50.0)	<0.01
Target vessel				0.23
	LAD	38 (55.9)	11 (45.8)	
	Diagonal	0 (0)	1 (4.2)	
	LCx	8 (11.8)	2 (8.3)	
	OM	3 (4.4)	1 (4.2)	
	RCA	14 (20.6)	9 (37.5)	
	Not available	5 (7.4)	0 (0.0)	

Values are median (interquartile range) or number (%).

Neovascularization volume is measured in 1000 x *μ*m^3^/mm. Macrophage accumulation is measured in macrophage grade/mm.

TCFA: thin-cap fibroatheroma; MLA: minimum luminal area; LAD: left anterior descending; LCx: left circumflex; OM: obtuse marginal; RCA: right coronary artery.

Neovascularization was significantly more frequent in TCFA than non-TCFA lesions (87.5% *versus* 65.7% respectively, p=0.04). Accordingly, TCFA had a markedly larger volume of neovascularization than non-TCFA plaques (92.2 [31.8-239.7] *versus* 23.0 [0.0-99.0] 1000 x *μ*m^3^/mm respectively, p<0.01).

At multivariate logistic analysis, both neovascularization volume and the degree of macrophage accumulation remained independently associated with the presence of TCFA (Table 3).

**Table 3:** Univariate and multivariate logistic regression analyses of predictors of thin-cap fibroatheromas

Variables	Univariate	p value	Multivariate	p value
	OR (95%CI)		OR (95%CI)	
Neovascularization volume	1.004 (1.001-1.006)	<0.01	1.003 (1.000-1.005)	0.03
Macrophage accumulation	1.420 (1.202-1.678)	<0.01	1.388 (1.169-1.647)	<0.01

OR: odds ratio; 95%CI: 95% confidence interval.

**Figure 1:**
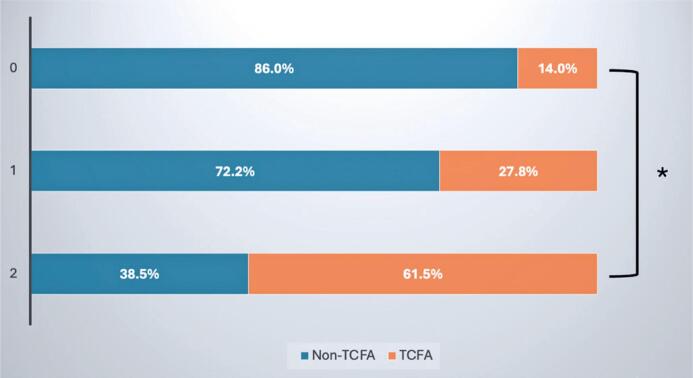
Presence of thin-cap fibroatheroma according to plaque inflammation classification

To further explore such an association, the dataset was divided according to the highest tercile of neovascularization volume (≥87.2 x 1000*μ*m^3^/mm) and macrophage accumulation score (≥4.6). Plaques with low levels of both neovascularization and macrophages were classified as TCFA in 14% of cases. Conversely, TCFA was present in 61.5% of segments with high macrophagic and neovascularization content (Figure 1).

* p<0.01.

Categories are as follows: 0= both neovascularization volume and macrophage accumulation below the cut-off values; 1= either neovascularization or macrophage accumulation above cut-off values; 2= both neovascularization volume and macrophage accumulation above cut-off values. Cut-off values are ≥87.6 x 1000*μ*m^3^/mm for neovascularization volume and ≥4.6 for macrophage accumulation score.

TCFA: thin-cap fibroatheroma.

## DISCUSSION

This is a retrospective study investigating the association between plaque-level inflammation and TCFA using OCT. Larger neovascularization volumes and higher degrees of macrophage accumulation were associated with TCFA, particularly when both features were present.

The formation process of TCFA is deeply dependent on local inflammation and angiogenesis, with macrophages exerting a catabolic effect on the fibrous cap of atherosclerotic plaques while neovessels nourish plaque growth.^(6,20)^

Macrophages secrete several collagen-breaking enzymes and cytokines, degrading the fibrous cap and making these lesions prone to rupture. It has been demonstrated that local macrophage density is associated with thinner fibrous caps and larger lipidic pools, both of which are essential features of TCFA.^(12,20–23)^

Neovascularization is a physiological response to the ischemic injury induced by atherosclerosis on the vessel wall, providing oxygen to the thickened intima. Neo-formed vessels are prone to intra-plaque hemorrhages, which cause accelerated plaque growth and enlargement of the lipidic core, as well as providing continuous antigenic stimulus by amplifying local inflammatory response and macrophage activation.^(6,24–26)^

The link between local inflammatory response and plaque vulnerability has also been explored in *in-vivo* studies using OCT. Raffel et al.^(14)^ used a complex method to assess fibrous cap macrophage density derived from raw OCT data. The authors found that lesions with TCFA presented higher density of macrophages when compared to non-TCFA lesions (7.35 *versus* 4.97, p<0.001), with a significant inverse linear correlation between fibrous cap thickness and macrophage density (r= −0.547, p=0.001). Likewise, Amano et al.^(10)^ found that lesions with neovascularization presented TCFA and macrophage infiltration more frequently than those without it (58% *versus* 11%, p<0.001 and 61% *versus* 26%, p=0.004, respectively), all of which are congruent with our own results. However, our study offers some new insights, as we investigated not only the presence of neovascularization or macrophage accumulation, but rather how their intensity was more frequently associated with TCFA. Thus, it is reasonable to assume that plaques with both features very intensely present have a higher inflammatory activity, which translates into thinner fibrous caps.

This study has several limitations. First, it is a single-center, retrospective study with a relatively small sample, and we did not include patients with sub-optimal image quality, which may have inadvertently resulted in a selection bias. Second, OCT has a limited penetration depth, which may have hindered our capacity to detect neovascularization and macrophages, particularly behind lipid-rich plaques, which are an integral part of TCFA. Third, our assessment was limited to a single point in time, and patients did not undergo follow-up OCT to assess plaque evolution. Despite all these limitations, our results were highly significant and are based on sound physiopathology. Nevertheless, this study is merely hypothesis-generating, and prospective studies with follow-up OCT imaging could provide more robust evidence regarding this subject.

## CONCLUSION

The present study provides a potentially unique insight into plaque-level intensity of inflammatory activity as assessed with optical coherence tomography. The volume of neovascularization and the intensity of macrophage accumulation were not only related to the presence of thin-cap fibroatheromas, but their combination was highly predictive of plaque vulnerability.

## References

[1] Libby P, Theroux P (2005). Pathophysiology of coronary artery disease. Circulation.

[2] Hansson GK (2005). Inflammation, atherosclerosis, and coronary artery disease. N Engl J Med.

[3] Ridker PM, Everett BM, Thuren T, MacFadyen JG, Chang WH, Ballantyne C, Fonseca F, Nicolau J, Koenig W, Anker SD, Kastelein JJP, Cornel JH, Pais P, Pella D, Genest J, Cifkova R, Lorenzatti A, Forster T, Kobalava Z, Vida-Simiti L, Flather M, Shimokawa H, Ogawa H, Dellborg M, Rossi PRF, Troquay RPT, Libby P, Glynn RJ, CANTOS Trial Group (2017). Antiinflammatory Therapy with Canakinumab for Atherosclerotic Disease. N Engl J Med.

[4] Welt FG, Rogers C (2002). Inflammation and restenosis in the stent era. Arterioscler Thromb Vasc Biol.

[5] Romero ME, Yahagi K, Kolodgie FD, Virmani R (2015). Neoatherosclerosis From a Pathologist's Point of View. Arterioscler Thromb Vasc Biol.

[6] Hayden MR, Tyagi SC (2004). Vasa vasorum in plaque angiogenesis, metabolic syndrome, type 2 diabetes mellitus, and atheroscleropathy: a malignant transformation. Cardiovasc Diabetol.

[7] Jang IK, Tearney GJ, MacNeill B, Takano M, Moselewski F, Iftima N (2005). In vivo characterization of coronary atherosclerotic plaque by use of optical coherence tomography. Circulation.

[8] Yabushita H, Bouma BE, Houser SL, Aretz HT, Jang IK, Schlendorf KH (2002). Characterization of human atherosclerosis by optical coherence tomography. Circulation.

[9] Di Vito L, Agozzino M, Marco V, Ricciardi A, Concardi M, Romagnoli E (2015). Identification and quantification of macrophage presence in coronary atherosclerotic plaques by optical coherence tomography. Eur Heart J Cardiovasc Imaging.

[10] Amano H, Koizumi M, Okubo R, Yabe T, Watanabe I, Saito D (2017). Comparison of Coronary Intimal Plaques by Optical Coherence Tomography in Arteries With Versus Without Internal Running Vasa Vasorum. Am J Cardiol.

[11] Jang IK, Bouma BE, Kang DH, Park SJ, Park SW, Seung KB (2002). Visualization of coronary atherosclerotic plaques in patients using optical coherence tomography: comparison with intravascular ultrasound. J Am Coll Cardiol.

[12] Kolodgie FD, Burke AP, Farb A, Gold HK, Yuan J, Narula J (2001). The thin-cap fibroatheroma: a type of vulnerable plaque: the major precursor lesion to acute coronary syndromes. Curr Opin Cardiol.

[13] Virmani R, Kolodgie FD, Burke AP, Farb A, Schwartz SM (2000). Lessons from sudden coronary death: a comprehensive morphological classification scheme for atherosclerotic lesions. Arterioscler Thromb Vasc Biol.

[14] Raffel OC, Tearney GJ, Gauthier DD, Halpern EF, Bouma BE, Jang IK (2007). Relationship between a systemic inflammatory marker, plaque inflammation, and plaque characteristics determined by intravascular optical coherence tomography. Arterioscler Thromb Vasc Biol.

[15] Virmani R, Burke AP, Kolodgie FD, Farb A (2003). Pathology of the thin-cap fibroatheroma: a type of vulnerable plaque. J Interv Cardiol.

[16] Prati F, Jenkins MW, Di Giorgio A, Rollins AM (2011). Intracoronary optical coherence tomography, basic theory and image acquisition techniques. Int J Cardiovasc Imaging.

[17] Tearney GJ, Regar E, Akasaka T, Adriaenssens T, Barlis P, Bezerra HG, Bouma B, Bruining N, Cho JM, Chowdhary S, Costa MA, de Silva R, Dijkstra J, Di Mario C, Dudek D, Falk E, Feldman MD, Fitzgerald P, Garcia-Garcia HM, Gonzalo N, Granada JF, Guagliumi G, Holm NR, Honda Y, Ikeno F, Kawasaki M, Kochman J, Koltowski L, Kubo T, Kume T, Kyono H, Lam CC, Lamouche G, Lee DP, Leon MB, Maehara A, Manfrini O, Mintz GS, Mizuno K, Morel MA, Nadkarni S, Okura H, Otake H, Pietrasik A, Prati F, Räber L, Radu MD, Rieber J, Riga M, Rollins A, Rosenberg M, Sirbu V, Serruys PW, Shimada K, Shinke T, Shite J, Siegel E, Sonoda S, Suter M, Takarada S, Tanaka A, Terashima M, Thim T, Uemura S, Ughi GJ, van Beusekom HM, van der Steen AF, van Es GA, van Soest G, Virmani R, Waxman S, Weissman NJ, Weisz G, International Working Group for Intravascular Optical Coherence Tomography (IWG-IVOCT) (2012). Consensus standards for acquisition, measurement, and reporting of intravascular optical coherence tomography studies: a report from the International Working Group for Intravascular Optical Coherence Tomography Standardization and Validation. J Am Coll Cardiol.

[18] Tahara S, Morooka T, Wang Z, Bezerra HG, Rollins AM, Simon DI (2012). Intravascular optical coherence tomography detection of atherosclerosis and inflammation in murine aorta. Arterioscler Thromb Vasc Biol.

[19] Takano M, Yamamoto M, Inami S, Murakami D, Ohba T, Seino Y (2009). Appearance of lipid-laden intima and neovascularization after implantation of bare-metal stents extended late-phase observation by intracoronary optical coherence tomography. J Am Coll Cardiol.

[20] Libby P (2002). Inflammation in atherosclerosis. Nature.

[21] Lee RT, Libby P (1997). The unstable atheroma. Arterioscler Thromb Vasc Biol.

[22] Libby P, Ridker PM, Maseri A (2002). Inflammation and atherosclerosis. Circulation.

[23] Henein MY, Vancheri S, Longo G, Vancheri F (2022). The Role of Inflammation in Cardiovascular Disease. Int J Mol Sci.

[24] de Boer OJ, van der Wal AC, Teeling P, Becker AE (1999). Leucocyte recruitment in rupture prone regions of lipid-rich plaques: a prominent role for neovascularization?. Cardiovasc Res.

[25] O’Brien KD, McDonald TO, Chait A, Allen MD, Alpers CE (1996). Neovascular expression of E-selectin, intercellular adhesion molecule-1, and vascular cell adhesion molecule-1 in human atherosclerosis and their relation to intimal leukocyte content. Circulation.

[26] Kumamoto M, Nakashima Y, Sueishi K (1995). Intimal neovascularization in human coronary atherosclerosis: its origin and pathophysiological significance. Hum Pathol.

